# Healthcare providers’ perspectives of disrespect and abuse in maternity care facilities in Nigeria: a qualitative study

**DOI:** 10.1007/s00038-019-01306-0

**Published:** 2019-10-31

**Authors:** Joy Orpin, Shuby Puthussery, Barbara Burden

**Affiliations:** 1 Maternal and Child Health Research Centre, Institute for Health Research, University of Bedfordshire, Putteridge Bury, Hitchin Road, Luton, Bedfordshire LU2 8LE UK; 2 School of Health Care Practice, University of Bedfordshire, Luton, Bedfordshire UK

**Keywords:** Respectful care, Disrespect and abuse, Healthcare providers, Health facilities, Maternity care, Nigeria

## Abstract

**Objectives:**

To explore healthcare providers’ perspectives of disrespect and abuse in maternity care and the impact on women’s health and well-being.

**Methods:**

Qualitative interpretive approach using in-depth semi-structured interviews with sixteen healthcare providers in two public health facilities in Nigeria. Interviews were audio-recorded, transcribed, and analysed thematically.

**Results:**

Healthcare providers’ accounts revealed awareness of what respectful maternity care encompassed in accordance with the existing guidelines. They considered disrespectful and abusive practices perpetrated or witnessed as violation of human rights, while highlighting women’s expectations of care as the basis for subjectivity of experiences. They perceived some practices as well-intended to ensure safety of mother and baby. Views reflected underlying gender-related notions and societal perceptions of women being considered weaker than men. There was recognition about adverse effects of disrespect and abuse including its impact on women, babies, and providers’ job satisfaction.

**Conclusions:**

Healthcare providers need training on how to incorporate elements of respectful maternity care into practice including skills for rapport building and counselling. Women and family members should be educated about right to respectful care empowering them to report disrespectful practices.

## Introduction

Disrespect and abuse (D&A) in childbirth are defined as ‘interactions or facility conditions that local consensus deems to be humiliating or undignified, and those interactions or conditions that are experienced as or intended to be humiliating or undignified’ (Freedman et al. 2014). Women’s experiences of D&A in maternity care have been reported from many countries in various forms such as being ignored, shouted at, slapped, and abandoned to deliver a child alone (Kruk et al. 2014; Lukasse et al. 2015; Sethi et al. 2017). These practices have broadly been categorised into discrimination, physical abuse, non-consented care, non-dignified care, abandonment or neglect, non-confidential care, and detention (Bowser and Hill 2010). The drivers of D&A are multifaceted including health system level factors such as poor provider supervision and training, lack of resources, poor policies, and poor nursing and midwifery management, as well as maternity service user related factors, such as lack of women empowerment and education (Bohren et al. 2015; Bowser and Hill 2010; Shimoda et al. 2018).

Experiences of D&A can result in women’s avoidance and/or delayed use of health facilities (Bowser and Hill 2010; Kujawski et al. 2015; Patel et al. 2015; Peca and Sandberg 2018). It could also have adverse effects on women’s reproductive lives; for example, a lack of desire to have children, fear of getting pregnant and childbirth, and choice of the mode of childbirth (Lukasse et al. 2015; Schroll et al. 2013). There are efforts to address the issue of D&A worldwide, including the development and implementation of policy guidelines (White Ribbon Alliance 2011). According to a recent WHO policy, ‘every woman has the right to the highest attainable standard of health, which includes the right to dignified, respectful health care’ (World Health Organization 2015). The Respectful Maternity Care Charter has outlined the links between D&A and maternal health rights within human rights such as the right to be free from harm and ill treatment, and the right to dignity and respect (White Ribbon Alliance 2011). Studies have indicated interventions to reduce disrespectful and abusive maternity care (Abuya et al. 2015; Austad et al. 2017; Ratcliffe et al. 2016). For example, Kujawski et al. (2017) report on an intervention to reduce the D&A of women in two facilities in Tanzania using the *Staha* intervention. The intervention aimed to enable health system stakeholders and local community members to analyse their personal experiences of D&A and reflect on practices to minimise it. They implemented the intervention among women who had facility-based childbirth, and its evaluation revealed that women’s self-report of disrespectful care reduced substantially (Kujawski et al. 2017).

Qualitative and quantitative studies have focussed on women’s experiences and perceptions of D&A during childbirth (Asefa and Bekele 2015; McMahon et al. 2014; Warren et al. 2017), but very few have explored this issue from the provider perspective (Burrowes et al. 2017; Rominski et al. 2017; Shimoda et al. 2018). In one study from Ghana, midwifery students reported witnessing and participating in various forms of D&A during childbirth and provided justifications for their perpetration of such practices (Rominski et al. 2017).

Nigeria accounts for over 14% of global maternal mortality (Izugbara et al. 2016), with about 35% of women giving birth in health facilities including 20% in public facilities and the remaining women in private settings (National Population Commission 2009). The previous studies have documented high prevalence rates of D&A in Nigeria ranging between 23.7 and 98%, but provider perspectives on D&A remain sparsely explored (Bohren et al. 2016, 2017). In a study exploring women’s and providers’ acceptability of physical restraints, verbal abuse, slapping, and refusal to help a woman in labour in Abuja, Nigeria, both women and providers reported witnessing and experiencing scenarios of mistreatment (Bohren et al. 2016). The participants reported factors such as health systems constraints such as the infrastructural facilities, poor provider attitudes, and the behaviour of women as contributors to mistreatment (Bohren et al. 2017). Though these studies offered insights into various forms of mistreatment and their acceptability, they were limited in focus and the scope, and more evidence is needed to develop a comprehensive understanding to inform policy, practice, and interventions.

Our study aimed to examine: (1) how maternity care providers perceived D&A of women during maternity care in Benue State, Nigeria; and (2) how maternity care providers viewed its impact on women’s health and well-being and their utilisation of maternity services.

## Methods

This study adopted a qualitative interpretive approach (Green and Thorogood 2014), and the data were derived from the second phase of a qualitative phenomenological study that aimed to explore women’s experiences and healthcare providers’ perspectives of D&A in maternity care facilities. The findings from the first phase of the study on women’s experiences have been reported elsewhere (Orpin et al. 2018).

### Study setting

The study was conducted in two public health facilities in Benue State, Nigeria—Benue State University Teaching Hospital (BSUTH) and Epidemiological Unit, Benue State Ministry of Health (EBSMH). BSUTH is a tertiary facility that provides healthcare to people in Benue State and Nigeria. It has about 350-bed capacity over 23 clinical departments including Obstetrics & Gynaecology (O&G) and Family Medicine, with a staff strength of over 800 (Benue State University Teaching Hospital 2018). The EBSMH is an outpatient facility providing postnatal services including immunisation to new-borns (Government of Benue State 2016). These health facilities were purposively selected because they are centrally situated and easily accessible by the people of Benue State and provide a wide range of maternal and neonatal services in the region.

### Participants and recruitment

Healthcare providers who deliver maternity care services and have been working for more than 1 year at any health facility were eligible for participation. A purposive sampling method was used to recruit maternity service providers (Green and Thorogood 2014). Permission to carry out the study was obtained from the Head of Obstetrics and Gynaecology (O&G) in BSUTH and the Chief Medical Director in EBSMH who assigned a medical doctor and an administrative staff member to assist the first author with the recruitment process.

All the participants were given an invitation letter and a participant information sheet and completed a consent form before participation. Twelve healthcare providers participated from BSUTH and four from EBSMH. The sample size was determined based on data saturation (Fusch and Ness 2015).

### Data collection

The first author conducted sixteen individual face-to-face semi-structured interviews with maternity care providers. Two healthcare providers from BSUTH participated in the pilot study to test the flexible interview guide, and the interview guide did not require any changes. We did not include the findings from the pilot study in the main study due to lack of consent from participants. The interview guide consisted of questions developed based on the previous studies and findings from the first phase on women’s experiences of D&A in maternity care (Orpin et al. 2018). The interviewer used probes as necessary to further explore a given response or for clarity (Green and Thorogood 2014). The interview started with the participants’ descriptions of their roles as maternity care providers and their general views on respectful maternity care. Participants were then probed to describe incident(s) they had witnessed/experienced where they felt the care provided was disrespectful and abusive; why they thought such incidents occurred; and the perceived impact of D&A on women.

Participants provided demographic information such as age and job titles before the interview in a self-recorded interview cover sheet. The researcher assured the women of confidentiality and clarified any queries both at the start and at the end of the interviews (Green and Thorogood 2014). The interviews lasted between 20 and 72 min and were conducted between September and October 2017. The first author (JO) who has a background in public health and experience of qualitative research conducted all the interviews in the participant’s office or in a quiet room free from interruptions where privacy was assured. The interviews were audio-recorded with permission from the participants.

### Data analysis

The first author (JO) transcribed all the audio-recorded interviews, and the second author (SP) checked the transcripts for accuracy and consistency. Using a thematic process in NVivo version 11, the transcripts were inductively coded. The analytic process applied involved reading the transcripts repeatedly for familiarisation, generation of initial codes, identification, naming and revising themes, and presenting the findings thematically (Braun and Clarke 2006). The first and second authors developed the overarching themes together, and the third author reviewed the themes for accuracy and consistency. The researchers used excerpts from the transcripts to illustrate the emerged themes along with number codes to identify the participants and to protect their identities (Green and Thorogood 2014).

The health research ethics committee of the University of Bedfordshire, UK, and the Government of Benue State, and BSUTH Ethics Committee approved this study.

## Results

Sixteen healthcare providers from various backgrounds and roles participated in the study including: consultants (O&G) (*n* = 4), medical doctors (*n* = 4), nurse–midwives (*n* = 5), chief nursing officers (*n* = 2), and community health extension worker (*n* = 1). The participants included both females and males; aged between 36 and 56 years old; and the years of work experience ranged between 8 and 33 years (Table 1). Five themes emerged from the data: (1) meanings of respectful maternity care; (2) subjectivity of woman-specific experiences; (3) ‘no harm intended’; (4) societal perceptions—‘women are weaker vessels’; and (5) potential impact of disrespect and abuse.

**Table 1:** Sociodemographic characteristics of healthcare providers participated in the study in Nigeria (2017)

Healthcare provider	Age	Gender	Tribe	Religion	Marital status	Job title	Number of years working in the role	Number of years of experience as a healthcare provider
HCP 1	40–49	F	Tiv	Christianity	Married	Chief nursing officer	8	22
HCP 2	50–59	F	Igala	Christianity	Married	Chief nursing officer	25	25
HCP 3	50–59	F	Etulo	Christianity	Married	Nurse–midwife	30	30
HCP 4	40–49	F	Yoruba	Christianity	Married	Nurse–midwife	5	10
HCP 5	30–39	F	Idoma	Christianity	Married	Nurse–midwife	9	9
HCP 6	50–59	M	Tiv	Christianity	Married	Consultant O&G	4	19
HCP 7	40–49	F	Tiv	Christianity	Married	Community health worker	5	13
HCP 8	40–45	M	Etsako	Christianity	Married	Consultant O&G	9	18
HCP 9	50–59	F	Tiv	Christianity	Married	Nurse–midwife	8	33
HCP 10	30–39	Fs	Tiv	Christianity	Married	Medical doctor	10	10
HCP 11	30–39	F	Idoma	Christianity	Married	Consultant O&G	7	13
HCP 12	40–49	M	Kaka	Christianity	Married	Medical doctor	8	10
HCP 13	30–39	M	Idoma	Christianity	Married	Medical doctor	6	8
HCP 14	50–59	M	Tiv	Christianity	Married	Consultant O&G	2	27
HCP 15	40–49	F	Tiv	Christianity	Married	Nurse–midwife	13	13
HCP 16	40–49	M	Idoma	Christianity	Married	Medical doctor	10	10

*HCP* Healthcare provider

### Meanings of respectful maternity care

This theme reflected participants’ descriptions of practices they considered either as respectful maternity care or as violation of women’s rights. The majority of the participants mentioned that the period of pregnancy is a vulnerable time, and women should be given good quality care in a humane manner to ensure safe childbirth and satisfactory care experiences. The participants viewed that pregnant women have the rights to be provided with information and informed consent, women-centred care, and respect for their wishes:

“Respectful maternity care is giving respect and giving the expected care […] the patient has the right to be given that health care that she is demanding for, and in giving that healthcare service, the patients also need her respect.” (HCP 3, Nurse-midwife)

“I would put it in a way that to sum it up, it’s all about a woman that is pregnant delivering with the best of care, delivering a healthy baby and a healthy mother going home satisfied.” (HCP 8, Consultant O&G)

All the participants described D&A as the failure to provide the expected good quality care to women during pregnancy and childbirth. Some healthcare providers reported that practices and actions such as not providing information, preferential treatment, creating uncomfortable care environments, carrying out procedures without consent, and forms of verbal abuse were a violation of a woman’s right to be treated with respect:

“If her consent is not sought before procedures or treatments are given. You just give it to her as if she has no say.” (HCP11, Consultant O&G)

“If you don’t make the environment tidy, clean and comfortable, that is disrespect of the patient.” (HCP11, Consultant O&G)

“If you do not counsel a patient properly, that is an abuse of the patient’s right.” (HCP 6, Consultant O&G)

### Subjectivity of woman-specific experiences

This theme reflected how the healthcare providers viewed D&A as a subjective experience for every woman that is influenced by individual perceptions of the care received in the maternity facilities and the attributed meanings to actions from healthcare staff:

“…it varies with patients. […] some will say they shouted at me there; they asked me to go back home; they did this and that to me. In that same place other women will go and be happy that they treated them well.” (HCP 15, Nurse-midwife)

“I think this disrespect should be individualised. Different clients attach a different meaning to what you do for them in the hospitals.” (HCP 1, Chief Nursing Officer)

A few of the participants linked women’s antenatal and childbirth care expectations to their experiences of D&A. They mentioned that some women tended to have specific care expectations, for example, of being cared for ‘as soon they walk’ into a health facility. When such expectations are unmet, they were likely to regard their experience as disrespectful and abusive:

“When a patient comes in, that woman comes in with the impression that immediately I get to the hospital, the midwife will attend to me.” (HCP 4, Nurse-midwife)

“By the time they come to the hospital, they will expect that everything should go their way and then they will not be satisfied with anything you do for them.” (HCP 2, Chief Nursing Officer)

### ‘No harm intended’

Healthcare providers perceived their actions and practices as not intended to bring any harm to the women. Those who expressed this view felt some practices and actions such as holding down a woman’s legs, slapping, and shouting were carried out with good intentions to ensure a safe pregnancy and childbirth.

Almost all the participants mentioned that the safety of a mother and her unborn child were of paramount importance when caring for women from the period of pregnancy to childbirth, and some actions may not be intended to disrespect women:

“Well, as a well-trained midwife, you will not just intentionally want to disrespect or abuse a woman who is in labour for any reason, but sometimes you just have to consider the safety of the unborn child and the mother first.” (HCP 2, Chief Nursing Officer)

Participants described instances they felt the safety of the mother and unborn child were at risk due to the lack of ‘cooperation’ from the woman, where in measures, like holding down women’s legs, were perceived as necessary and sometimes used:

“There are times when women will close their legs during labour or when the baby is coming. At that time the only thing you are thinking is how she will give birth to her child well […] nurses will hold their legs so that they can push out the baby.” (HCP 16, Medical Doctor)

Other actions like shouting at women who appear to be uncooperative during childbirth were carried out to gain compliance:

“If a woman is really in the second stage of labour […] and close her legs, they can even kill the baby. […] You will go the extra mile to make sure […] even if it means you shouting…you have a safe delivery.” (HCP 4, Nurse-midwife)

The healthcare providers also suggested that D&A sometimes result from unintended and uncontrollable circumstances encountered in health facilities such as overcrowding, inadequate medical supplies and equipment, shortage of staff, and lack of proper training in respectful maternity care. They mentioned, for example, that women who have had their childbirth in the open maternity wards would not have their privacy protected which can be seen as D&A:

“Some of the women when they come here to deliver their babies, the labour room is not covered, like no screens but they are okay and happy because they deliver their babies safely.” (HCP 5, Nurse-midwife)

As a further indication of circumstances outside of their immediate control, some of the participants mentioned that they had not received any training specific to respectful maternity care apart from what they had learned in nursing and midwifery and medical schools:

“We do not have those workshops on the issue of respect of women in our hospital here, though we have workshops that involve things on safe motherhood but nothing directly on respectful care.” (HCP 16, Medical Doctor)

### Societal perceptions—‘women are weaker vessels’

Healthcare providers described how societal perceptions influence women’s experiences of disrespectful and abusive care. A few of them, mostly men, stated how women are regarded in the African society as ‘weaker’ and ‘less privileged’ than men which leads to their voices being unheard. This was perceived to result in some healthcare providers treating women as they are not important:

“… Unfortunately we see women as… weaker vessels […] in African society. They say a woman is supposed to be seen and not heard… the healthcare providers tend to see them as second-class citizens or third-class citizen and they tend to look down on them.” (HCP 8, Consultant O&G)

“In our African culture and even in other countries of the world, women do not enjoy many privileges like men […] sometimes you find some doctors do treat women in a certain way that is not respectful because of this cultural belief that men are above women.” (HCP 13, Medical Doctor)

### Potential impact of disrespect and abuse

The participants described the potential impact of D&A on women’s health and well-being, subsequent use of health facilities, and the job satisfaction for healthcare providers. More than half of them stated that the experience of D&A could result in poor health care-seeking behaviour as it could prevent or delay access to health facilities because women no longer have trust in the system:

“…the woman would have lost confidence in the health facility and the health system and it may prevent her from accessing health services in that same facility” (HCP 3, Chief Nursing Officer).

Some healthcare providers felt that the women with negative experiences may inform others and discourage them from accessing health facilities. They also reported that home births with unskilled birth attendants or family members who can understand and provide the needed support may become the preferred choice for successive childbirths:

“She will […] prefer to have her baby at home where family members that understand her culture and belief will respect her…” (HCP1, Chief Nursing Officer).

More than half of the participants felt that the experience of D&A could adversely affect women’s health and well-being resulting in a wide range of obstetric complications like prolonged labour, and postpartum haemorrhage and these complications could be contributing to maternal and infant deaths:

“It could contribute to maternal and prenatal mortality and morbidity which we are all struggling to reduce, but disrespecting and abusing women is indirectly contributing to it” (HCP1, Chief Nursing Officer).

“If you didn’t provide the information she needs, she can lose the pregnancy.” (HCP 12, Medical Doctor)

A few participants indicated that an experience D&A could leave a negative recollection of the event that may lead to the fear of childbirth, low self-esteem, and postnatal depression:

“So, it will leave an unpleasant memory and she is going to approach further childbearing with fear because of her experience.” (HCP11, Consultant O&G)

“I feel that when women are traumatised from the process of such disrespect and abuse, it definitely affects their self-esteem… it may even contribute to some of the baby blues and depression that women have.” (HCP10, Medical Doctor)

Leaving women with a negative care experience was also seen to have an indirect impact on the job satisfaction for healthcare providers:

“You don’t have the job satisfaction because you are not giving the care that you should give.” (HCP 10, Medical Doctor)

## Discussion

This study sought to explore the perceptions of healthcare providers on D&A of women during maternity care and its impact on their health, well-being, and utilisation of maternity services in Benue State, Nigeria. The findings indicated five interrelated themes that included: meanings of respectful maternity care; subjectivity of woman-specific experiences; ‘no harm intended’; gendered societal perceptions; and the potential impact of disrespect and abuse. At times, these themes crossed over and overlapped in the participant accounts. The healthcare providers in our study appeared to have an awareness of components of respectful maternity care within the human rights’ perspective such as maintaining confidentiality and privacy and provision of information (Shakibazadeh et al. 2018; White Ribbon Alliance 2011). Similar to findings in the previous studies, the healthcare providers recognised incidents of disrespectful and abusive care in various stages of maternity care (Asefa et al. 2018; Burrowes et al. 2017; Shimoda et al. 2018; Warren et al. 2015). Their accounts indicated that many of the rights as outlined in the Respectful Maternity Care Charter (White Ribbon Alliance 2011) such as the right to information and informed consent, and respect for choices and preferences, were violated in practice.

The healthcare providers perceived D&A as a subjective experience for women accessing maternity facilities, and the women’s experience of disrespectful care is often based on their personal feelings and expectations of care. This would suggest that women whose maternity care expectations are not met are likely to feel disrespected and abused (Bruggemann et al. 2012). It also highlights the need for woman-centred and holistic care approaches such as the intrapartum care model for a positive maternity experience (World Health Organization 2018). The healthcare providers also felt certain practices and actions such as holding down women’s legs and shouting were carried out with good intentions to gain their cooperation to ensure the women have a safe pregnancy and childbirth. The providers’ desire to help women and their prioritisation of safe childbirth were seen as the motivating factors to engage in some practices that may in fact be viewed as D&A.

Additionally, the challenges within the health facilities such as overcrowding sometimes lead to the perception that the practices were disrespectful and abusive. This concurred with the women’s views as reflected in the first phase of our study (Orpin et al. 2018). Other authors also reported similar findings (Bohren et al. 2016; Rominski et al. 2017), although d’Oliveira et al. (2002) have argued that service providers often abuse women deliberately.

A campaign to include respectful maternity care in all Nigerian health facilities was endorsed by the National Council for Health who recognised the Respectful Maternity Care Charter and approved respectful care as the standard procedure for healthcare delivery (McConville 2014). The healthcare providers in our study, however, had not received any training on respectful practices apart from what they had learned in nursing, midwifery, or medicals schools. This shows the need for action by Nigerian stakeholders to ensure healthcare providers are adequately trained about respectful maternity practices including elements of rapport building and counselling skills as part of their curricula in nursing, midwifery, or medicals schools along with timely refresher trainings to mitigate the issue.

The healthcare providers also attributed D&A to the cultural belief that women are weaker than men by portraying them as less privileged compared to men. The previous research has shown that Nigerian culture is predominantly patriarchal in nature and upholds male dominance (Makama 2013), and as a result, the rights of women are often violated in and outside of the home. This highlights the need for strategies such as campaigns in local community centres and media platforms that focus on the adverse effect of such beliefs on women, their families, and the wider society. Sustained community engagement could bring about changes in social norms (Marston et al. 2013). This could possibly promote wider societal change and can enhance respectful care for women in Nigerian healthcare facilities. The gender-based explanations for D&A also indicate the need for appropriate interventions focused on notions of gender equality for maternity care professionals.

Our study found that the healthcare providers recognised the impact D&A can have on women’s health and well-being and their use of health facilities including the impact on women’s reproductive lives, sexuality and desire for children (Schroll et al. 2013), and their own job satisfaction. Poor health-seeking behaviour following experiences of D&A was also seen to be a consequence (Bohren et al. 2014; Burrowes et al. 2017; D’Ambruoso et al. 2005) which could lead to obstetric complications, and subsequently morbidity and mortality (National Population Commission 2014). This would suggest that D&A could be a contributor to the low uptake of maternity care services and high rates of maternal mortality in Nigeria, alongside other factors that hinder timely access to services. Further research should explore the specific links between D&A and the uptake of maternity services and/or the rates of maternal mortality in Nigeria.

This is one of the few qualitative studies that have explored healthcare providers’ perspectives of D&A in maternity care in Nigeria, and the first study conducted in Benue State. Our findings add to the existing body of evidence on D&A on professional perceptions about the meaning and the impact of D&A during all stages of maternity care including antenatal, childbirth, and postpartum. However, the qualitative nature of the research methodology may limit the generalisation of the findings. The use of purposive sampling allowed the capture of perspectives from a range of healthcare providers enabling a comprehensive exploration of the topic and transferability and representativeness of the findings (Green and Thorogood 2014). The individual semi-structured interviews enabled the participants to freely provide their responses, though their work commitment may have infringed on the duration of the interviews.

Large scale quantitative and mixed method studies with representative sample sizes would help to ascertain if the findings in our study can be generalised to Nigeria or other African countries. Future research could benefit from including perspectives and experiences of policymakers and other non-clinical staff members in maternity care settings. Understanding the cultural, social, and environmental factors associated with disrespectful and abusive practices and drivers for behaviour change are also crucial to inform the development of tailored intervention strategies to address D&A.

### Conclusion

The accounts from healthcare providers in our study indicated understanding about what respectful maternity care encompassed, though the application in practice appeared insufficient based on their self-reports of D&A. Our findings reflect the need for sensitising healthcare providers through refresher training on how to incorporate elements of respectful care into everyday practice. Due to the multi-cultural nature of the country, training activities should take into account components of cultural competence. Strengthening health system capacity by employing adequate number of healthcare providers, especially in areas where there is an unmet need, along with improvement in infrastructure facilities, will also reduce D&A practices interpreted to be caused by issues like overcrowding in facilities and shortage of providers. The underlying societal perceptions point to the need for awareness campaigns and educational interventions at wider sociopolitical and community levels including educating women and men about rights to respectful care.

## Abbreviations

D&A: Disrespect and abuse
HCP: Healthcare providers
O&G: Obstetrics and gynaecology
WHO: World Health Organisation
